# Identification of “Multiple Components-Multiple Targets-Multiple Pathways” Associated with Naoxintong Capsule in the Treatment of Heart Diseases Using UPLC/Q-TOF-MS and Network Pharmacology

**DOI:** 10.1155/2016/9468087

**Published:** 2016-03-31

**Authors:** Xianghui Ma, Bin Lv, Pan Li, Xiaoqing Jiang, Qian Zhou, Xiaoying Wang, Xiumei Gao

**Affiliations:** ^1^State Key Laboratory of Modern Chinese Medicine, Tianjin University of Traditional Chinese Medicine, Tianjin 300193, China; ^2^College of Traditional Chinese Medicine, Tianjin University of Traditional Chinese Medicine, Tianjin 300193, China

## Abstract

Naoxintong capsule (NXT) is a commercial medicinal product approved by the China Food and Drug Administration which is used in the treatment of stroke and coronary heart disease. However, the research on the composition and mechanism of NXT is still lacking. Our research aimed to identify the absorbable components, potential targets, and associated pathways of NXT with network pharmacology method. We explored the chemical compositions of NXT based on UPLC/Q-TOF-MS. Then, we used the five principles of drug absorption to identify absorbable ingredients. The databases of PharmMapper, Universal Protein, and the Molecule Annotation System were used to predict the main targets and related pathways. By the five principles of drug absorption as a judgment rule, we identified 63 compositions that could be absorbed in the blood in all 81 chemical compositions. Based on the constructed networks by the significant regulated 123 targets and 77 pathways, the main components that mediated the efficacy of NXT were organic acids, saponins, and tanshinones. Radix Astragali was the critical herbal medicine in NXT, which contained more active components than other herbs and regulated more targets and pathways. Our results showed that NXT had a therapeutic effect on heart diseases through the pattern “multiple components-multiple targets-multiple pathways.”

## 1. Introduction

Naoxintong capsule (NXT) is a commercial medicinal product approved by the China Food and Drug Administration which is widely used in the treatment of stroke and coronary heart disease. NXT contains 16 Chinese herbal medicines (Table 1). NXT exerts significant therapeutic effects and has high safety for stroke recovery in the clinical setting [1]. Recent studies showed that NXT could reduce the infarct size of acute myocardial infarction (AMI) patients by improving vascular endothelial function [2]. Long-term administration of NXT was also reported to alleviate inflammation, reduce the recurrence of angina pectoris, and decrease the incidence of ACS attack in borderline lesion coronary heart disease patients [3]. Some studies investigated the mechanisms of NXT in vitro or in vivo. NXT was reported to protect against atherosclerosis through its lipid-lowering activity [4] and to reduce the expression of iNOS mRNA and the NO level in the vessel wall to benefit the treatment of atherosclerosis [5]. NXT also protected cardiomyoblasts against H_2_O_2_-induced oxidative injury [6]. Although some mechanisms of NXT have been reported, existing studies on unilateral factors and single targets could not demonstrate the complex mechanisms of NXT, a herbal prescription with 16 medicines which is prescribed for the treatment of complex diseases like cardiovascular and cerebrovascular diseases.

With the prominence of network pharmacology in system biology, this distinct and novel approach to the study of complicated analytical systems is becoming more widely known and more frequently used in the field of drug research. The functions of network pharmacology include uncovering the functions of traditional Chinese medicines (TCMs), providing deeper insights into and scientific evidence for TCMs, and identifying TCMs as scientifically proven. Here, we attempt to explore the mechanism of NXT using this method.

In the current study, based on the use of UPLC/Q-TOF-MS to investigate the involved components, we aimed to analyse the absorbable components of NXT, to identify potential targets and associated pathways using the network pharmacology method, and to systematically discuss the mechanism of NXT in the treatment of heart diseases.

## 2. Material and Methods

### 2.1. Prediction of Components

#### 2.1.1. Sample Preparation

NXT was obtained from HezeBuchang Pharmaceutical Co., Ltd. (Heze, China). Deionized water was prepared from aqua distillate using a Milli-Q system (Millipore, Bedford, MA, USA). Analytical grade methanol was purchased from Merck (Darmstadt, Germany). We dissolved 1 g of NXT powder in 10 mL of 75% analytical grade methanol and subjected the mixture to ultrasonic extraction for 30 min. We then brought the solution to room temperature and obtained the supernatant as a capture reagent. The sample was filtered using a 0.22 *μ*m microporous membrane before UPLC analysis.

#### 2.1.2. UPLC/Q-TOF-MS

We used a Waters Acquity UPLC System (Waters Co., USA) furnished with a photodiode array detector for the analysis. The sample was diluted on a Waters Acquity UPLC BEH C18 column (2.1 mm × 100 mm, 1.7 *μ*m). UV detection was achieved at 190–400 nm. The system was controlled using the MassLynx version 4.1 software (Waters Co.). The gradient duration program for A (UPLC-grade acetonitrile) and B (water with 0.1% formic acid) was performed as follows: 2% A from 0 min to 3 min, 10% to 50% A from 3 min to 12 min, 50% to 63% A from 12 min to 18 min, 63% to 83% A from 18 min to 21 min, 83% to 84% A from 21 min to 22 min, 84% to 87% A from 22 min to 26 min, 87% to 90% A from 26 min to 28 min, 90% to 95% A from 28 min to 31 min, 95% to 100% A from 31 to 33 min, 100% to 100% A from 33 to 35 min, and 100% to 2% A from 35 min to 37 min. The flow rate was maintained at 0.4 mL/min, and the column temperature was maintained at 30°C.

The components of NXT were identified using a Waters Q-TOF Premier with an electrospray ionization (ESI) system (Waters MS Technologies, Manchester, UK). The ESI-MS spectra were acquired at both negative and positive ion voltages. The capillary voltage was set to 2.5 kV for the negative mode and to 3.0 kV for the positive mode. The sample cone voltage was set to 30 V, and the source temperature was 110°C. High-purity nitrogen was used as the nebulization and auxiliary gas. The nebulization gas was set to 600 L/h, the cone gas was set to 50 L/h, and the desolation temperature was 350°C. The Q-TOF Premier acquisition rate was 0.1 s, and there was a 0.02 s interscan delay. Argon, which was used as the collision gas, was maintained at a pressure of 5.3 × 10^−5^ Torr. The instrument was operated with the first resolving quadruple in a wide pass mode (100 Da–1500 Da). Leucineen kephalinamide acetate was used as the lock mass ([M − H]^−^ = 553.2775, [M + H]^+^ = 555.2931).

### 2.2. Calculation and Prediction of Absorbable Components

First, we determined the structural formulas of the chemical components that were identified in compound NXT from the Chemical Book website and used the Chemdraw software to draw these formulas. Then, we imported these structural formulas into the Online SMILES Translator and Structure File Generator (http://cactus.nci.nih.gov/translate/) to obtain the smiles format. Finally, we input the smiles format of the chemical components into the Molispiration Smiles website (http://www.molinspiration.com/cgi-bin/properties) to calculate the prediction parameters of drug absorption. According to the five principles of drug absorption, if a component was subject to the following provisions of the corresponding parameters, it could be identified as an absorbable component: hydrogen bond donor (the number of hydrogen atoms attached to the O and N) *n*OHNH ≤ 5; relative molecular mass MW ≤ 500; fat water partition coefficient miLog*P* ≤ 5; and hydrogen bond acceptor (the number of O and N) *n*ON ≤ 10.

### 2.3. Prediction and Screening of Targets

Using the software of Chembio3D Ultru12.0, we transformed the structure of the absorbed components into the sdf structure format. Then, to predict the possible targets, we imported the components into the public network server of the target database of the efficacy group PharmaMapper website (http://59.78.96.61/pharmmapper/) to perform reverse docking. We selected the top 10 targets for subsequent study.

### 2.4. Prediction and Screening of Pathways

We imported the obtained targets into the Bio database (http://bioinfo.capitalbio.com/mas3/) and then screened for pathways that met the criterion of *P* < 0.01.

### 2.5. Construction of Network

According to the screening pathways with their corresponding targets and components, we created a component-target-pathway illustration using Cytoscape. Then, according to the main selected targets, we drew a target-composition diagram.

## 3. Results

### 3.1. UPLC/Q-TOF-MS Analysis

**Figure 1:**
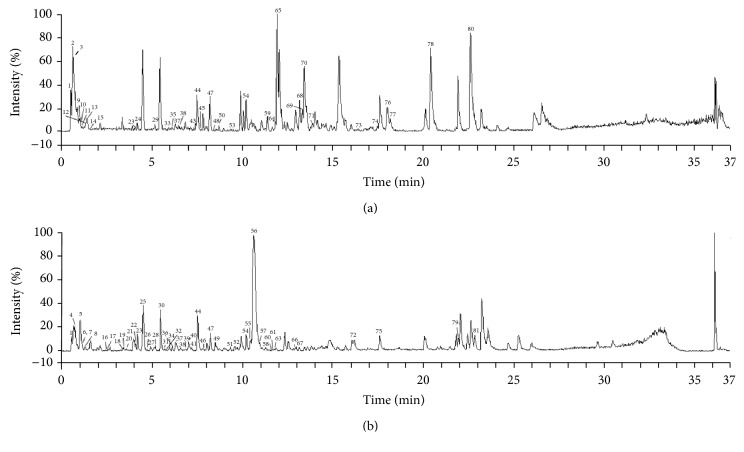
UPLC/Q-TOF-MS analysis of NXT. (a) Chromatograms of NXT in positive ion mode. (b) Chromatograms of NXT in negative ion mode.

We analysed the chemical components of NXT using ultraperformance liquid chromatography combined with quadrupole time-of-flight mass spectrometry. Because different chemical components had better responses in different modes, MS data were obtained in both positive ion mode (Figure 1(a)) and negative ion mode (Figure 1(b)). MS data in (+/−) ESI modes and the identification results for the constituents in NXT were presented in Table 2. In all 16 herbs from NXT, no related component in Myrrha and Hirudo was found.

### 3.2. Absorption Parameters of Components

**Figure 2:**
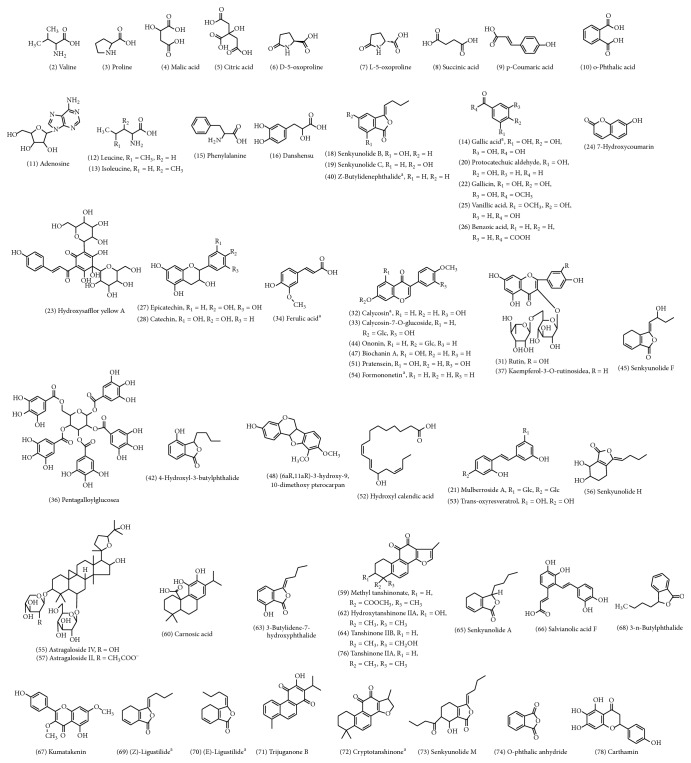
Structures of 63 absorbable components.

Using a computer prediction method to calculate the identified compounds of NXT, we obtained absorption parameters that could determine whether the chemical compositions could be absorbed.  Table 3 showed the specific absorption parameters of all of the components. The data indicated that there were a total of 63 chemical compositions (Figure 2) that met the five principles of drug absorption. As shown, 7 glycosides were identified. Although the relative molecular masses of those compounds were greater than 500, they could also be absorbed, because those compounds could be divided into two parts, including aglycones which mainly mediated drug efficacy and sugar chains in the body. So we could import these glycosides' aglycones into PharmMapper to obtain the relevant parameters. The results showed that both of these components were consistent with the five principles of drug absorption, so we considered that these 7 chemical compositions could be absorbed in the body.

### 3.3. Potential Targets and Pathways

By importing 63 chemical compositions that were predicted to be absorbable into the PharmMapper database for directional docking, we obtained a total of 123 targets. We then imported these targets into the Molecule Annotation System and obtained 77 pathways regulated by NXT with highly significant differences, from which we chose the top 40 pathways that met the criterion of *P* < 0.01 (Table 4). A total of 34 targets were related to these top 40 pathways, and HRAS, MAP2K1, and MAPK14 were associated with most of these pathways, so we considered these factors to be the main targets. As shown in Table 4, NFAT and hypertrophy of the heart (transcription in the broken heart) ranked first among these pathways.

In Table 5, these top 40 pathways were classified into 5 categories, which included pathways associated with heart diseases and blood vessels, metabolism, cell cycle (with proliferation and apoptosis), immunity, and other pathways. By classifying these pathways, we accessed and marked the corresponding medicinal materials of NXT (Table 5). In the pathways associated with heart diseases and blood vessels, RCX, RSM, and FC were the most important. In the regulation of metabolism, RA, RSM, and RCX showed diametrical effect. All the herbs except Semen Persicae (SP) were related metabolism pathways due to the current research. RA, RSM, RCX, and FC could regulate the pathways about cell cycle, proliferation, and apoptosis. Some other important pathways were also affected by some herbs like RA, RSM, and RCX, for example, Insulin Signaling Pathway and p38 MAPK Signaling Pathway.

### 3.4. Pharmacology Network of NXT

**Figure 3:**
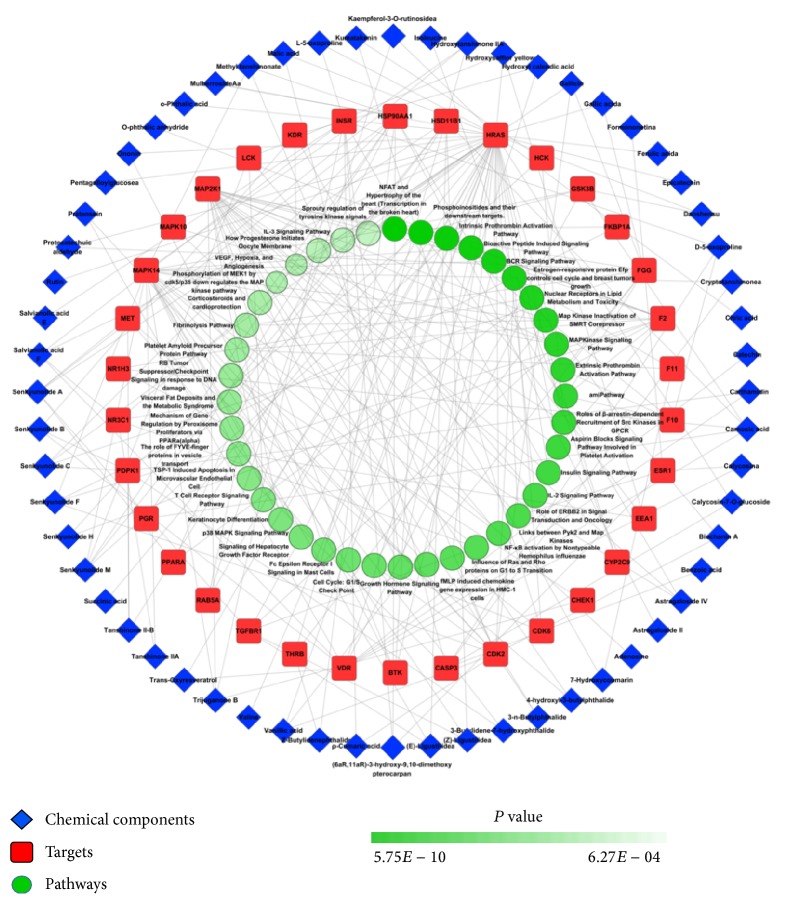
Pharmacology network of the “components-targets-pathways” regulated by NXT.

Using the Cytoscape software, we constructed a pharmacology network of NXT (Figure 3), which showed us the relationships of the top 40 pathways, targets, and chemical components. We obtained preliminary understanding of the mechanism of NXT through this network.

**Figure 4:**
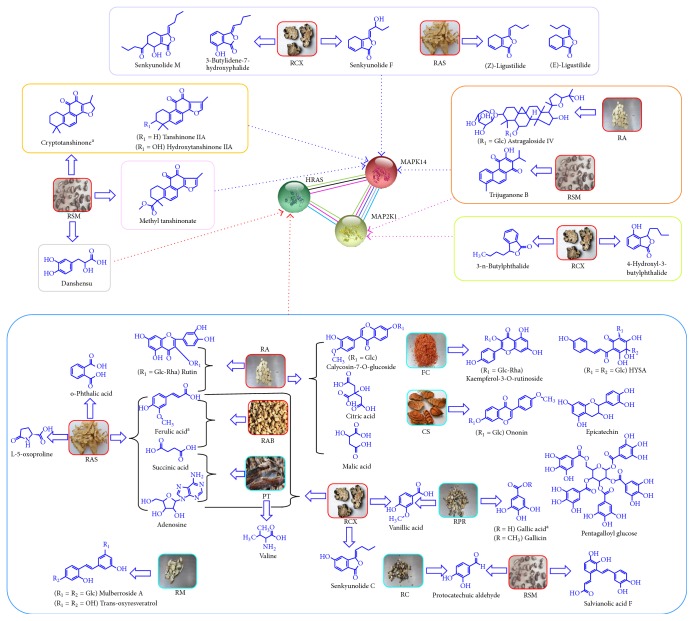
Network of major targets of NXT with corresponding compounds.

In this research, we found three major targets of NXT: HRAS, MAP2K1, and MAPK14, which were involved in most regulated pathways. By Figure 4, based on illustration of the main targets with their corresponding compounds, we found the most effective ingredients of NXT were organic acids, saponins, and tanshinones. The main sources of organic acids were RA, RCX, RAS, and RAB. The saponins were mainly derived from RA. Meanwhile, tanshinones were mainly concentrated in RSM.

## 4. Discussion

The burden of cardiovascular and circulatory disease is becoming more and more serious, with cerebrovascular disease (CBD) and ischemic heart disease being the most serious [7]. As the causes of cardiovascular disease (CVD) and CBD are complicated, the symptoms of these diseases are also very diverse. NXT is commonly used during clinical treatment of CVD and CBD, and the effect of this drug is remarkable. Although complex traditional Chinese medicine has great significance for the treatment of complex diseases, some questions such as the material basis and the potential mechanisms remain unanswered.

Our study successfully predicted absorbable chemical compositions of NXT. These constituents primarily included ferulic acid, succinic acid, astragaloside IV, and tanshinone IIA. Ferulic acid, which is derived primarily from RA, RCX, RAS, and RAB, is reported to act as an angiogenic agent that augments angiogenesis, which is critical in ischemic diseases, such as myocardial infarction and stroke [8]. Succinic acid has been demonstrated to activate Akt phosphorylation to inhibit apoptosis and necrosis caused by cardiomyocyte hypoxia/reoxygenation [9]. Previous studies demonstrated that astragaloside IV could protect the heart through NO-dependent mechanism [10]. NO has been confirmed to prevent the mitochondrial permeability transition pore from opening [11]. During early reperfusion, it can prevent the heart from reperfusion injury by inhibiting the opening of the mitochondrial permeability transition pore [12]. Tanshinone IIA also has cardioprotective effects, such as protection of cardiomyocytes from oxidative stress-triggered damage [13]. These reports were consistent with our results.

In addition to active ingredients, we also successfully predicted drug targets of NXT. The major targets were HRAS, MAP2K1, and MAPK14. The HRAS gene encodes the GTPase HRas, which is an enzyme known as transforming protein p21 [14]. With the ability to increase the effects of growth factor, HRas plays an important role in regulating the growth, differentiation, and death of endothelial cells [15]. The MAP2K1 gene encodes an enzyme named dual specificity mitogen-activated protein kinase kinase 1, and MAPK14 encodes p38-*α*. Both of these factors are closely related to inflammation and p38-*α* is also associated with cardiac hypertrophy via p38 MAPK activity in the heart. In addition, p38-*α* has been recognized as an isoenzyme of cardiovascular importance [16].

Among the numerous identified pathways, NFAT and hypertrophy of the heart (transcription in the broken heart) were ranked first. Nuclear factor of activated T-cells (NFAT) transcription factors, which have four different isoforms, plays crucial roles in the regulation of gene expression during heart development [17]. The isoforms NFATc3 and NFATc4 are involved in hypertrophic development, while NFATc1 plays a key role in cardiac development [18]. The dephosphorylation of NFATs can promote calcineurin regulating immune response genes [19]. Via compensatory hypertrophy, the heart adapts to persistent stress conditions, but, over time, dysfunction and myocardial failure evolve [20]. Like NFAT and hypertrophy of the heart (transcription in the broken heart), most of these pathways are involved in the formation and regulation of cardiovascular disease, such as nuclear receptors in lipid metabolism and toxicity. Nuclear receptors include a superfamily of ligand-dependent transcription factors that regulate genetic networks that control cell growth, development, and metabolism. Regulating nuclear receptors is beneficial for patients with metabolic diseases, such as cardiovascular disease, due to the requirement for balance among a number of pathways for normal metabolic control [21]. These studies confirmed the validity of our study.

From the above results, we also found the different significances of the total of 16 herbs in NXT. According to Chinese Pharmacopoeia 2015, the content of RA in NXT is 66 g, which is 2-3 times the content of any other herb in the whole prescription. It was reported that RA was the monarch drug of NXT and played a key role in improving the immune system, invigorating blood circulation, and the condition of myocardial ischemia and hypoxia [22]. Our study found that RA contained a lot of effective components, organic acids, and saponins and was critical source of the main active components of NXT. Through the comparison of the herbs involved in the top 40 pathways, RA was also proved to be the most important. In the top 40 pathways regulated by NXT, RA was involved in 33 pathways. Some other herbs, such as RSM, RCX, FC, and RAS, were also the important contents in the whole prescription of NXT.

The network pharmacology method used in this study is a novel methodology based on the construction of multilayer networks of disease phenotype-gene-drug to predict drug targets in a holistic manner and promote efficient drug discovery [23]. This method represents a breakthrough in comparison to the traditional herbal medicine research pattern “gene-target-disease” and initiates the new pattern “multiple genes-multiple targets-complex diseases” [24]. By this method, we proved that RA was the critical ingredient mainly involved in the regulation of metabolism and immunity in NXT. RAS was a major herb that regulated cell growth. RSM, RCX, and FC also played important roles in regulation of heart disease, blood vessels, and others. The results indicated that NXT, a complex prescription in the treatment of complex diseases, played a therapeutic effect through multiple targets and multiple pathways. This was the first study to investigate the mechanism of NXT using this method, and we successfully predicted the main targets and pathways, providing a foundation for further research. This method has important value for the study of complex drugs and should be applied in future studies.

## 5. Conclusion

The main components that mediated the efficacy of NXT were organic acids, saponins, and tanshinones. Radix Astragali was the critical herbal medicine in NXT, which contained more active components than others and regulated more targets and pathways. NXT had a therapeutic effect on the treatment of heart diseases through the pattern “multiple components-multiple targets-multiple pathways.”

## Figures and Tables

**Table 1:** Sixteen Chinese traditional medical herbs of NXT.

Abbreviation	Medicinal herbs	Original plants	Content (g)^*∗*^
RA	Radix Astragali	*Astragalus membranaceus *(Fisch.) Beg. var. *mongholicus* (Bge.) Hsiao or *A. membranaceus* (Fisch.) Bge.	66
RPR	Radix Paeoniae Rubra	*Paeonia lactiflora *Pall. or *P. veitchii *Lynch	27
RSM	Radix Salviae Miltiorrhizae	*Salvia miltiorrhiza *Bge.	27
RAS	Radix Angelicae Sinensis	*Angelica sinensis (Oliv) Diels.*	27
RCX	Rhizoma Chuanxiong	*Ligusticum chuanxiong *Hort.	27
SP	Semen Persicae	*Prunus persica* (L.) Batsch or *Prunusdavidiana* (Carr.) Franch.	27
FC	Flos Carthami	*Carthamus tinctorius* L.	13
FK	Frankincense	*Boswellia carterii Birdw.*	13
MRH	Myrrha	*Commiphora myrrha *Engl.	13
CS	Caulis Spatholobi	*Spatholobus suberectus *Dunn	20
RAB	Radix Achyranthis Bidentatae	*Achyranthes bidentata* Bl. or *Cyathula officinalis* Kuan	27
RC	Ramulus Cinnamomi	*Cinnamomum cassia* Presl	20
RM	Ramulus Mori	*Morus alba* L.	27
PT	Pheretima	*Pheretima aspergillum *(E. Perrier) or *Pheretima vulgaris* Chen. or* Pheretima guillelmi* (Michaelsen) or *Pheretima pectinifera* Michaelsen	27
SCP	Scorpio	*Buthus martensii* Karsch	13
HRD	Hirudo	*Whitmania pigra* Whitman or *Hirudo nipponica* Whitman or *Whitmania acranulata* Whitman	27

^*∗*^ *Note*. The content of 16 Chinese traditional medical herbs of NXT came from Chinese Pharmacopoeia 2015.

**Table 2:** MS data in (+/−) ESI modes and the identification results for the constituents in NXT.

Peak number	RT (min)	Identification	Mode	MS (*m*/*z*)	Composition	Herbal source
1	0.647	Arginine	Pos/Neg	174.2024	C_6_H_14_N_4_O_2_	PT
2	0.702	Valine	Pos	117.1478	C_5_H_11_NO_2_	PT
3	0.721	Proline	Pos	115.1331	C_5_H_9_NO_2_	PT
4	0.776	Malic acid	Neg	134.0911	C_4_H_6_O_5_	RA
5	1.053	Citric acid	Neg	192.1286	C_6_H_8_O_7_	RA
6	1.201	D-5-oxoproline	Neg	129.1174	C_5_H_7_NO_3_	RAS
7	1.201	L-5-oxoproline	Neg	129.1174	C_5_H_7_NO_3_	RAS
8	1.275	Succinic acid	Neg	118.0910	C_4_H_6_O_4_	RAS, RAB, PT
9	1.294	*ρ*-Coumaric acid	Pos	164.1601	C_9_H_8_O_3_	RAS
10	1.310	o-Phthalic acid	Pos	166.1294	C_8_H_6_O_4_	RAS
11	1.312	Adenosine	Pos	267.2403	C_10_H_13_N_5_O_4_	RAS, PT, RCX
12	1.331	Leucine	Pos	131.1688	C_6_H_13_NO_2_	PT
13	1.460	Isoleucine	Pos	131.1688	C_6_H_13_NO_2_	RAB
14	1.589	Gallic acid^a^	Neg	170.1207	C_7_H_6_O_5_	RPR
15	2.199	Phenylalanine	Pos	165.1874	C_9_H_11_NO_2_	FC
16	2.459	Danshensu	Neg	198.1701	C_9_H_10_O_5_	RSM
17	2.606	Palmitic acid	Neg	256.3380	C_16_H_32_O_2_	RAS, FC, RA, SCP
18	3.438	Senkyunolide B	Neg	204.2374	C_12_H_12_O_3_	RCX
19	3.456	Senkyunolide C	Neg	204.2374	C_12_H_12_O_3_	RCX
20	3.600	Protocatechuic aldehyde	Neg	138.1185	C_7_H_6_O_3_	RSM, RC
21	3.974	Mulberroside A^a^	Neg	568.5277	C_26_H_32_O_14_	RM
22	4.122	Gallicin	Neg	184.1453	C_8_H_8_O_5_	RPR
23	4.230	Hydroxysafflor yellow A	Pos/Neg	612.5364	C_27_H_32_O_16_	FC
24	4.232	7-Hydroxycoumarin	Pos	162.1457	C_9_H_6_O_3_	RM
25	4.565	Vanillic acid	Neg	168.1459	C_8_H_8_O_4_	RCX, RPR
26	4.694	Benzoic acid	Neg	122.1209	C_7_H_6_O_2_	RPR
27	4.935	Epicatechin	Neg	290.2674	C_15_H_14_O_6_	CS
28	5.157	Catechin	Neg	290.2674	C_15_H_14_O_6_	RPR
29	5.212	Albiflorin	Pos	480.4653	C_23_H_28_O_11_	RPR
30	5.730	Quercetin-7-O-glucoside	Neg	464.3754	C_21_H_20_O_12_	FC
31	5.952	Rutin	Neg	610.5203	C_27_H_30_O_16_	RA
32	5.970	Calycosin^a^	Neg	284.2679	C_16_H_12_O_5_	RA
33	5.988	Calycosin-7-O-glucoside	Pos	446.4075	C_22_H_22_O_10_	RA
34	5.989	Ferulic acid^a^	Neg	194.1815	C_10_H_10_O_4_	RA, RCX, RAS, RAB
35	6.321	Paeoniflorin^a^	Pos	480.466	C_23_H_28_O_11_	RPR
36	6.358	Pentagalloylglucose^a^	Neg	940.68	C_41_H_32_O_26_	RPR
37	6.413	Kaempferol-3-O-rutinoside^a^	Pos/Neg	594.5179	C_27_H_30_O_15_	FC
38	6.654	3,5-Di-O-caffeoylquinic acid^a^	Pos/Neg	516.4573	C_25_H_24_O_12_	CS
39	6.987	Dicaffeoylquinic acid	Neg	516.1275	C_25_H_24_O_12_	RCX
40	7.042	Z-Butylidenephthalide^a^	Neg	188.2259	C_12_H_12_O_2_	RCX
41	7.210	Salvianolic acid A	Neg	494.4578	C_26_H_22_O_10_	RSM
42	7.449	4-Hydroxyl-3-butylphthalide	Pos	206.2346	C_12_H_14_O_3_	RCX
43	7.540	Salvianolic acid B	Neg	718.6220	C_36_H_30_O_16_	RSM
44	7.688	Ononin	Pos	430.4107	C_22_H_22_O_9_	CS
45	7.763	Senkyunolide F	Pos	206.1017	C_12_H_14_O_3_	RCX, RAS
46	7.855	Salvianolic acid E	Neg	718.1512	C_36_H_30_O_16_	RSM
47	8.243	Biochanin A	Pos/Neg	284.2689	C_16_H_12_O_5_	CS
48	8.262	(6aR,11aR)-3-Hydroxy-9,10-dimethoxy pterocarpan	Pos	300.3107	C_17_H_16_O_5_	RA
49	8.594	N1-N5-(Z)-N10-(E)-tri-p-coumaroylspermidine	Pos	583.2703	C_34_H_37_N_3_O_6_	FC
50	8.740	Benzoylpaeoniflorin	Pos	584.5723	C_30_H_32_O_12_	RPR
51	9.518	Pratensein	Neg	300.0679	C_16_H_12_O_6_	RA
52	9.611	Hydroxyl calendic acid	Neg	294.4342	C_18_H_30_O_3_	SP
53	9.648	*Trans*-oxyresveratrol	Pos	244.2435	C_14_H_12_O_4_	RM
54	10.240	Formononetin^a^	Pos/Neg	268.2580	C_16_H_12_O_4_	RA
55	10.405	Astragaloside IV	Neg	784.4633	C_41_H_68_O_14_	RA
56	10.590	Senkyunolide H	Neg	220.2305	C_12_H_12_O_4_	RCX
57	10.978	Astragaloside II	Neg	826.4701	C_43_H_70_O_15_	RA
58	11.311	Soyasaponin I	Neg	942.5145	C_48_H_78_O_18_	RA
59	11.422	Methyl tanshinonate	Pos	338.1087	C_20_H_18_O_5_	RSM
60	11.588	Carnosic acid	Neg	332.4311	C_20_H_28_O_4_	RSM
61	11.644	Kaempferol-3-O-glucoside	Neg	448.3752	C_21_H_20_O_11_	FC
62	11.699	Hydroxytanshinone IIA	Pos	310.1199	C_19_H_18_O_4_	RSM
63	11.792	3-Butylidene-7-hydroxyphalide	Neg	204.2331	C_12_H_12_O_3_	RCX
64	11.921	Tanshinone II-B	Pos	310.1187	C_19_H_18_O_4_	RSM
65	12.198	Senkyunolide A	Pos	192.2516	C_12_H_16_O_2_	RCX
66	12.975	Salvianolic acid F	Neg	314.0735	C_17_H_14_O_6_	RSM
67	13.196	Kumatakenin	Neg	314.3359	C_17_H_14_O_6_	RA
68	13.233	3-n-Butylphthalide	Pos	190.2356	C_12_H_14_O_2_	RCX
69	13.474	(Z)-ligustilide^a^	Pos	190.2109	C_12_H_14_O_2_	RAS
70	13.483	(E)-ligustilide^a^	Pos	190.2109	C_12_H_14_O_2_	RAS
71	13.917	Trijuganone B	Pos	280.1107	C_18_H_16_O_3_	RSM
72	16.098	Cryptotanshinone^a^	Neg	296.3642	C_19_H_20_O_3_	RSM
73	16.394	Senkyunolide M	Pos	278.1565	C_16_H_22_O_4_	RCX
74	17.503	O-Phthalic anhydride	Pos	148.0207	C_8_H_4_O_3_	FC
75	17.614	Chlorogenic acid^a^	Neg	354.3120	C_16_H_18_O_9_	CS
76	18.076	Tanshinone IIA	Pos	294.3430	C_19_H_18_O_3_	RSM
77	18.205	Angelicide	Pos	380.1917	C_24_H_28_O_4_	RCX
78	20.460	Carthamidin	Pos	288.2575	C_15_H_12_O_6_	FC
79	22.078	Linoleic acid	Neg	280.2387	C_18_H_32_O_2_	SP
80	22.659	Acetyl-11-keto-*β*-boswellic acid	Pos/Neg	512.7458	C_32_H_48_O_5_	FK
81	22.881	Oleanolic acid	Neg	456.3652	C_30_H_48_O_3_	RSM

“a” refers to the component has been verified by standard substance.

**Table 3:** Absorption parameters of the components.

Number	Compounds	MW	*n*ON	*n*OHNH	miLog*P*	Results
1	Arginine	174.204	6	7	−3.632	*✕*
2	Valine	117.15	3	3	−1.91	√
3	Proline	115.132	3	2	−1.723	√
4	Malic acid	134.087	5	3	−1.57	√
5	Citric acid	192.123	7	4	−1.983	√
6	D-5-oxoproline	129.115	4	2	−2.402	√
7	L-5-oxoproline	129.115	4	2	−2.402	√
8	Succinic acid	118.088	4	2	−0.655	√
9	*ρ*-Coumaric acid	164.160	3	2	1.43	√
10	o-Phthalic acid	166.132	4	2	1.034	√
11	Adenosine	267.245	9	5	−0.854	√
12	Leucine	131.175	3	3	−1.382	√
13	Isoleucine	131.175	3	3	−1.41	√
14	Gallic acid^a^	170.120	5	4	0.589	√
15	Phenylalanine	165.192	3	3	−1.231	√
16	Danshensu	198.174	5	4	−0.251	√
17	Palmitic acid	256.43	2	1	7.059	*✕*
18	Senkyunolide B	204.225	3	1	2.81	√
19	Senkyunolide C	204.225	3	1	2.574	√
20	Protocatechuic aldehyde	138.122	3	2	0.759	√
21	Mulberroside A^a^	568.528	14	10	−0.852	√
22	Gallicin	184.147	5	3	0.848	√
23	Hydroxysafflor yellow A	612.54	16	12	−4.12	√
24	7-Hydroxycoumarin	162.144	3	1	1.511	√
25	Vanillic acid	168.148	4	2	1.187	√
26	Benzoic acid	122.123	2	1	1.848	√
27	Epicatechin	290.271	6	5	1.369	√
28	Catechin	290.271	6	5	1.369	√
29	Albiflorin	480.466	11	5	−1.636	*✕*
30	Quercetin-7-O-glucoside	464.379	12	8	−0.104	*✕*
31	Rutin	610.521	16	10	−1.063	√
32	Calycosin^a^	284.267	5	2	2.377	√
33	Calycosin-7-O-glucoside	446.408	10	5	0.59	√
34	Ferulic acid^a^	194.186	4	2	1.249	√
35	Paeoniflorin^a^	480.466	11	5	0.044	*✕*
36	Pentagalloylglucose^a^	940.681	26	15	2.761	√
37	Kaempferol-3-O-rutinoside^a^	594.522	15	9	−0.574	√
38	3,5-Di-O-caffeoylquinic acid^a^	516.455	12	7	1.424	*✕*
39	Dicaffeoylquinic acid	516.46	12	7	1.21	*✕*
40	Z-Butylidenephthalide^a^	188.226	2	0	3.077	√
41	Salvianolic acid A	494.452	10	7	3.014	*✕*
42	4-Hydroxyl-3-butylphthalide	206.241	3	1	3.42	√
43	Salvianolic acid B	718.620	16	9	1.615	*✕*
44	Ononin	430.409	9	4	1.307	√
45	Senkyunolide F	206.24	3	1	1.72	√
46	Salvianolic acid E	718.62	16	10	2.83	*✕*
47	Biochanin A	284.267	5	2	2.804	√
48	(6aR,11aR)-3-Hydroxy-9,10-dimethoxy pterocarpan	300.31	5	1	2.546	√
49	N1-N5-(Z)-N10-(E)-tri-p-coumaroylspermidine	538.68	9	5	4.3	*✕*
50	Benzoylpaeoniflorin	584.574	12	4	2.472	*✕*
51	Pratensein	300.27	6	3	2.09	√
52	Hydroxyl calendic acid	294.435	3	2	4.93	√
53	Trans-Oxyresveratrol	244.246	4	4	2.723	√
54	Formononetin^a^	268.268	4	1	3.095	√
55	Astragaloside IV	784.98	14	9	1.21	√
56	Senkyunolide H	220.224	4	2	2.314	√
57	Astragaloside II	827.02	15	8	1.91	√
58	Soyasaponin I	943.13	18	11	1.7	*✕*
59	Methyl tanshinonate	338.36	5	0	0.93	√
60	Carnosic acid	332.440	4	3	4.603	√
61	Kaempferol-3-O-glucoside	448.380	11	7	0.125	*✕*
62	Hydroxytanshinone IIA	310.35	4	1	3.24	√
63	3-Butylidene-7-hydroxyphthalide	204.225	3	1	2.81	√
64	Tanshinone II-B	310.35	4	1	2.97	√
65	Senkyunolide A	192.258	2	0	3.521	√
66	Salvianolic acid F	314.29	6	5	2.33	√
67	Kumatakenin	314.29	6	2	2.98	√
68	3-n-Butylphthalide	190.242	2	0	3.483	√
69	(Z)-Ligustilide^a^	190.242	2	0	2.927	√
70	(E)-Ligustilide^a^	190.242	2	0	2.927	√
71	Trijuganone B	280.32	3	1	3.9	√
72	Cryptotanshinone^a^	296.366	3	0	3.83	√
73	Senkyunolide M	278.35	4	1	2.55	√
74	O-Phthalic anhydride	148.12	3	0	0.93	√
75	Chlorogenic acid^a^	354.311	9	6	−0.453	*✕*
76	Tanshinone IIA	294.350	3	0	4.158	√
77	Angelicide	380.48	4	0	5.73	*✕*
78	Carthamidin	288.255	6	4	1.649	√
79	Linoleic acid	280.45	2	1	6.86	*✕*
80	Acetyl-11-keto-*β*-boswellic acid	512.73	5	1	6.39	*✕*
81	Oleanolic acid	456.71	3	2	6.72	*✕*

*Note*. “√” means that component could be absorbed; “*✕*” means that component could not be absorbed.

“a” refers to the component has been verified by standard substance.

**Table 4:** Top 40 Biocarta pathways regulated by NXT (*P* < 0.01).

Rank	Pathway	Count	*P*-value	*q*-value	Gene
1	NFAT and hypertrophy of the heart (transcription in the broken heart)	6	5.75*E* − 10	3.58*E* − 09	HRAS; GSK3B; MAPK14; FKBP1A; F2; MAP2K1
2	Phosphoinositides and their downstream targets	5	1.39*E* − 09	8.47*E* − 09	GSK3B; PDPK1; BTK; RAB5A; EEA1
3	Intrinsic Prothrombin Activation Pathway	4	8.50*E* − 08	2.82*E* − 07	F10; FGG; F11; F2
4	Bioactive Peptide Induced Signaling Pathway	4	4.08*E* − 07	9.30*E* − 07	HRAS; MAPK14; F2; MAP2K1
5	BCR Signaling Pathway	4	4.88*E* − 07	1.08*E* − 06	HRAS; MAPK14; BTK; MAP2K1
6	Estrogen-responsive protein Efp controls cell cycle and breast tumors growth	3	6.40*E* − 07	1.34*E* − 06	CDK2; ESR1; CDK6
7	Nuclear receptors in lipid metabolism and toxicity	4	8.02*E* − 07	1.58*E* − 06	CYP2C9; VDR; NR1H3; PPARA
8	Map kinase inactivation of SMRT corepressor	3	1.53*E* − 06	2.48*E* − 06	THRB; MAPK14; MAP2K1
9	MAP Kinase Signaling Pathway	5	2.09*E* − 06	3.05*E* − 06	HRAS; MAPK10; MAPK14; TGFBR1; MAP2K1
10	Extrinsic Prothrombin Activation Pathway	3	2.99*E* − 06	4.05*E* − 06	F10; FGG; F2
11	amiPathway	3	5.17*E* − 06	6.40*E* − 06	F10; FGG; F2
12	Roles of *β*-arrestin-dependent recruitment of Src kinases in GPCR signaling	3	6.57*E* − 06	7.86*E* − 06	HRAS; HCK; MAP2K1
13	Aspirin blocks signaling pathway involved in platelet activation	3	8.19*E* − 06	9.49*E* − 06	HRAS; F2; MAP2K1
14	Insulin Signaling Pathway	3	2.03*E* − 05	2.03*E* − 05	HRAS; INSR; MAP2K1
15	IL-2 Signaling Pathway	3	2.37*E* − 05	2.29*E* − 05	HRAS; MAP2K1; LCK
16	Role of ERBB2 in signal transduction and oncology	3	2.37*E* − 05	2.29*E* − 05	HRAS; ESR1; MAP2K1
17	Links between Pyk2 and MAP kinases	3	2.74*E* − 05	2.45*E* − 05	HRAS; MAPK14; MAP2K1
18	NF-*κ*B activation by nontypeable Hemophilus influenzae	3	2.74*E* − 05	2.45*E* − 05	MAPK14; TGFBR1; NR3C1
19	Influence of Ras and Rho proteins on G1 to S transition	3	3.14*E* − 05	2.82*E* − 05	HRAS; CDK2; CDK6
20	fMLP induced chemokine gene expression in HMC-1 cells	3	3.14*E* − 05	2.82*E* − 05	HRAS; MAPK14; MAP2K1
21	Growth Hormone Signaling Pathway	3	3.14*E* − 05	2.82*E* − 05	HRAS; INSR; MAP2K1
22	Cell cycle: G1/S checkpoint	3	4.06*E* − 05	3.37*E* − 05	CDK2; GSK3B; CDK6
23	Fc epsilon receptor I signaling in mast cells	3	4.58*E* − 05	3.70*E* − 05	HRAS; BTK; MAP2K1
24	Signaling of hepatocyte growth factor receptor	3	6.40*E* − 05	4.89*E* − 05	HRAS; MET; MAP2K1
25	p38 MAPK signaling pathway	3	7.85*E* − 05	5.76*E* − 05	HRAS; MAPK14; TGFBR1
26	Keratinocyte differentiation	3	1.13*E* − 04	7.81*E* − 05	HRAS; MAPK14; MAP2K1
27	T cell receptor signaling pathway	3	1.13*E* − 04	7.81*E* − 05	HRAS; MAP2K1; LCK
28	TSP-1 induced apoptosis in microvascular endothelial cell	2	1.46*E* − 04	9.59*E* − 05	CASP3; MAPK14
29	The role of FYVE-finger proteins in vesicle transport	2	1.46*E* − 04	9.59*E* − 05	RAB5A; EEA1
30	Mechanism of gene regulation by peroxisome proliferators via PPARa(alpha)	3	1.82*E* − 04	1.15*E* − 04	HSP90AA1; NR1H3; PPARA
31	Visceral fat deposits and the metabolic syndrome	2	1.95*E* − 04	1.21*E* − 04	HSD11B1; NR3C1
32	RB tumor suppressor/checkpoint signaling in response to DNA damage	2	2.50*E* − 04	1.44*E* − 04	CDK2; CHEK1
33	Platelet Amyloid Precursor Protein Pathway	2	2.50*E* − 04	1.44*E* − 04	F11; F2
34	Fibrinolysis Pathway	2	3.12*E* − 04	1.77*E* − 04	FGG; F2
35	Corticosteroids and cardioprotection	2	3.12*E* − 04	1.77*E* − 04	HSP90AA1; NR3C1
36	Phosphorylation of MEK1 by cdk5/p35 downregulates the MAP kinase pathway	2	3.81*E* − 04	2.09*E* − 04	HRAS; MAP2K1
37	VEGF, hypoxia, and angiogenesis	2	5.38*E* − 04	2.79*E* − 04	HRAS; KDR
38	How progesterone initiates oocyte membrane	2	6.27*E* − 04	3.17*E* − 04	HRAS; PGR
39	IL-3 Signaling Pathway	2	6.27*E* − 04	3.17*E* − 04	HRAS; MAP2K1
40	Sprouty regulation of tyrosine kinase signals	2	6.27*E* − 04	3.17*E* − 04	HRAS; MAP2K1

**Table 5:** The herbs of NXT involved in the top 40 pathways.

Category	Pathway	NXT	RA	RPR	RSM	RAS	RCX	SP	FC	CS	RAB	RC	RM	PT
Pathway associated with heart diseases and blood vessels	NFAT and hypertrophy of the heart (transcription in the broken heart)	1	1	1	1	1	1	0	1	1	1	1	1	1
	Intrinsic Prothrombin Activation Pathway	1	0	1	1	1	1	0	1	0	0	0	1	0
	Extrinsic Prothrombin Activation Pathway	1	0	1	1	1	1	0	1	0	0	0	0	0
	Aspirin blocks signaling pathway involved in platelet activation	1	1	1	1	1	1	0	1	1	1	1	1	1
	TSP-1 induced apoptosis in microvascular endothelial cell	1	1	1	1	1	1	0	1	0	0	0	0	0
	Platelet Amyloid Precursor Protein Pathway	1	0	0	0	0	1	0	1	0	0	0	1	0
	Fibrinolysis Pathway	1	0	1	1	0	1	0	1	0	0	1	0	0
	Corticosteroids and cardioprotection	1	1	0	1	0	1	0	0	0	0	0	1	0
	VEGF, hypoxia, and angiogenesis	1	1	1	1	1	1	1	1	1	1	1	1	1

Pathway associated with metabolism	Nuclear receptors in lipid metabolism and toxicity	1	1	1	1	0	1	1	1	0	1	0	1	0
	Growth Hormone Signaling Pathway	1	1	1	1	1	1	0	1	1	1	1	1	1
	Visceral fat deposits and the metabolic syndrome	1	1	0	1	1	1	0	0	0	0	0	0	0

Pathway associated with immunity	*BCR Signaling Pathway*	1	1	1	1	1	1	0	1	1	1	1	1	1
	IL-2 Signaling Pathway	1	1	1	1	1	1	0	1	1	1	1	1	1
	fMLP induced chemokine gene expression in HMC-1 cells	1	1	1	1	1	1	0	1	1	1	1	1	1
	T Cell Receptor Signaling Pathway	1	1	1	1	1	1	0	1	1	1	1	1	1

Pathway associated with cell cycle, proliferation, and apoptosis	Phosphoinositides and their downstream targets	1	0	0	1	0	1	0	1	1	0	0	0	0
	Estrogen-responsive protein Efp controls cell cycle and breast tumors growth	1	1	1	1	1	1	0	0	1	0	0	0	0
	Map kinase inactivation of SMRT corepressor	1	1	1	1	1	1	0	1	0	0	0	0	0
	MAP Kinase Signaling Pathway	1	1	1	1	1	1	0	1	1	1	1	1	1
	Roles of *β*-arrestin-dependent recruitment of Src kinases in GPCR signaling	1	1	1	1	1	1	0	1	1	1	1	1	1
	Role of ERBB2 in signal transduction and oncology	1	1	1	1	1	1	0	1	1	1	1	1	1
	Links between Pyk2 and MAP kinases	1	1	1	1	1	1	0	1	1	1	1	1	1
	NF-*κ*B activation by nontypeable *Hemophilus influenzae*	1	1	0	1	1	1	0	1	0	0	0	0	0
	Influence of Ras and Rho proteins on G1 to S transition	1	1	1	1	1	1	0	1	1	1	1	1	1
	Cell cycle: G1/S checkpoint	1	1	1	1	1	1	0	0	1	0	0	0	0
	Fc epsilon receptor I signaling in mast cells	1	1	1	1	1	1	0	1	1	1	1	1	1
	Signaling of hepatocyte growth factor receptor	1	1	1	1	1	1	0	1	1	1	1	1	1
	Keratinocyte differentiation	1	1	1	1	1	1	0	1	1	1	1	1	1
	RB tumor Suppressor/checkpoint signaling in response to DNA damage	1	1	1	1	1	1	0	0	1	0	0	0	0
	IL-3 Signaling Pathway	1	1	1	1	1	1	0	1	1	1	1	1	1
	Sprouty regulation of tyrosine kinase signals	1	1	1	1	1	1	0	1	1	1	1	1	1

Other pathways	Bioactive Peptide Induced Signaling Pathway	1	1	1	1	1	1	0	1	1	1	1	1	1
	amiPathway	1	0	1	1	1	1	0	1	0	0	0	0	0
	Insulin Signaling Pathway	1	1	1	1	1	1	0	1	1	1	1	1	1
	p38 MAPK Signaling Pathway	1	1	1	1	1	1	0	1	1	1	1	1	1
	The role of FYVE-finger proteins in vesicle transport	1	0	0	0	0	0	0	1	0	0	0	0	0
	Mechanism of gene regulation by peroxisome proliferators via PPAR*α*	1	1	0	1	0	1	0	0	0	0	0	1	0
	Phosphorylation of MEK1 by cdk5/p35 downregulates the MAP kinase pathway	1	1	1	1	1	1	0	1	1	1	1	1	1
	How progesterone initiates oocyte membrane	1	1	1	1	1	1	1	1	1	1	1	1	1

*Note*. “1” means that the Chinese herbal medicine acts on the pathway while “0” means it does not. The pathways in each category are sorted by the significant differences in *P* value.

## Abbreviations

ACS: Acute coronary syndrome
AMI: Acute myocardial infarction
CBD: Cerebrovascular disease
CVD: Cardiovascular disease
ESI: Electrospray ionization
HRAS: Harvey rat sarcoma viral oncogene homolog
MAP2K1: Mitogen-activated protein kinase kinase 1
MAPK14: Mitogen-activated protein kinase 14
NFAT: Nuclear factor of activated T cells
NXT: Naoxintong capsule
TCMs: Traditional Chinese medicines
UPLC/Q-TOF-MS: Ultraperformance liquid chromatography/quadrupole time-of-flight mass spectrometry.
